# Involving Young People in Healthy Ageing: A Crucial Facet to Achieving the Decade of Healthy Ageing (2021–2030)

**DOI:** 10.3389/fpubh.2021.723068

**Published:** 2021-12-17

**Authors:** Brian L. H. Wong, Ines Siepmann, Apoorva Rangan, Omnia El-Omrani, Daniel Davis, Natalia Arias-Casais, Diah S. Saminarsih, David Gems

**Affiliations:** ^1^Medical Research Council Unit for Lifelong Health and Ageing at UCL, Department of Population Science and Experimental Medicine, UCL Institute of Cardiovascular Science, University College London, London, United Kingdom; ^2^Immunisation and Countermeasures Division, National Infection Service, Public Health England, London, United Kingdom; ^3^Young Professionals Programme, Association of Schools of Public Health in the European Region (ASPHER), Brussels, Belgium; ^4^Faculty of Health, Medicine, and Life Sciences, Maastricht University, Maastricht, Netherlands; ^5^Stanford University School of Medicine, Stanford, CA, United States; ^6^Faculty of Medicine, Ain Shams University, Cairo, Egypt; ^7^International Federation of Medical Students' Associations (IFMSA), Copenhagen, Denmark; ^8^Atlantes Global Observatory for Palliative Care, Institute for Culture and Society, University of Navarra, Pamplona, Spain; ^9^Office of the Director-General, World Health Organization, Genève, Switzerland; ^10^Department of Genetics, Evolution and Environment, Institute of Healthy Ageing, University College London, London, United Kingdom

**Keywords:** decade of health ageing, healthy ageing, intergenerational, youth education, ageism

## Introduction

In response to a rapidly ageing world population, the World Health Organisation (WHO) has proposed a *Decade of Healthy Ageing 2021–30* (hereafter: *the Decade*) to promote the health of over a billion people aged 60 and older (1). The COVID-19 pandemic has exposed faults in healthcare, equity, and economic safety affecting all humanity. According to the WHO's baseline report for *the Decade*, more than 40% of COVID-19-related deaths (up to 80% in some high-income countries) during 2020 are linked to long-term care facilities (2). Moreover, certain pandemic response measures (e.g., lockdowns) have exacerbated existing issues experienced by older adults around the world, such as depression, loneliness, and social isolation. COVID-19 has also added to the concerns of many young people about the future of health, healthcare, and social protection in old age, who must ask “in what kind of world do we want to live?” and “how do we want to age? (3)”

Healthy ageing is defined by the WHO as “developing and maintaining the functional ability that enables well-being in older age” (1). Recent comments and reports have called for more youth engagement in global health issues, including those which will impact them later in life (e.g., health governance) (4). Young people and youth are herein considered to be individuals between 15 and 29 years, which is inclusive of most young adults actively receiving education, entering the workforce, or early in their career. This commentary hereby aspires to reflect the vision of young health researchers and medical students in the discussion of *the Decade*'s four action areas. We argue that youth engagement should extend to ageing care, as youth perspectives are an important determinant of well-being in old age, both of youth themselves (in terms of their future selves) and how their actions affect older people in their communities.

Youth contribute to the barriers and stigmas encountered by older adults, make up a substantial portion of the health workforce engaged in older adult care, and are critical to effectively plan for a better and healthier future to foster intergenerational partnerships and prevent people from being left behind in the era of Universal Health Coverage (5). Innovative solutions, particularly surrounding social support and care, are necessary to address the otherwise unmanageable health needs of the ageing population (6). Meaningful engagement of young people in revolutionising ageing does not detract from the need to centre voices of older adults. Rather, it increases the scope of stakeholders involved and provides additional opportunities for positive actions to be realised. Younger and older adults are less visible and typically less represented in “all-age” initiatives. As such, bi-directional engagement and learning opportunities to promote such initiatives would be mutually beneficial.

## Changing Perceptions of Age and Ageing

The first action area of *the Decade* calls for governments to “change how we think, feel and act towards age and ageing” (1). The baseline report highlights how deeply rooted societies' negative perceptions are about older people. From a young age, children pick up cues from those around them about their culture's stereotypes and prejudices. Not only are these quickly internalised, but also build over time and develop into ageist stereotypes. These stereotypes are later employed to make inferences and guide one's feelings and behaviours towards people of different ages and towards oneself (7).

Immune system ageing has been a major contributor to older people dying at disproportionately high rates from COVID-19; the acceptance of disproportionately high deaths among the older population exemplifies the ageist principle of the expendability of older individuals. When trying to do something about ageism at individual, community, and institutional levels, governments should consider the body of evidence in favour of intergenerational programmes, in which individuals of varying ages collaborate, co-create, and engage in shared activities. The development of intergenerational social projects has been shown to advance the meaningful inclusion of the ageing population in communities, address negative attitudes towards ageing in youth and older adults, and model opportunities for youth to work across generations (8).

In an example of how intergenerational programmes can be mutually beneficial, medical students in Hong Kong accompanied older and terminally-ill patients in Grantham Hospital. By listening to the patients and leading a range of activities targeted at their emotional needs, students experienced development in terms of their empathy and communication skills (9). Participation in this programme enabled the students to better understand the patients' states of mind and more effectively alleviate the older patients' symptoms. Another example is Humanitas Deventer, an intergenerational care home in the Netherlands where students—in return for affordable housing—live alongside older neighbours and contribute to their social care (10). Students' attitudes and actions towards ageing shifted in response to their immersion in the care of older adults, and older individuals reported exiting the “sick role” often experienced in classic long-term care facilities (11). Other studies also support an association between informal intergenerational activities, such as occasional volunteering or friendships, and improved young people's attitudes towards working with older adults (8, 12).

The initiatives mentioned above often rely on students accessing formal education in health-related fields. Governments can support intergenerational initiatives by identifying additional young populations with the capacity to engage with older people as well as facilitating environments and opportunities to support these connections. In March 2021, the WHO released a guide on initiating conversations about ageism. This guide, which includes sections for children and adolescents, is a useful tool for facilitating discussions and acknowledgement surrounding ageism. To ensure the engagement of young people, adaptations should consider young people beyond adolescence (for example, university students, early career professionals) and include methods for them to encourage and start the conversation, not just be on the receiving end (13).

## Fostering Age-Friendly Environments

Second, *the Decade* asks governments to “ensure that communities foster the abilities of older people” (1). We encourage governments to foster youth engagement in near-term initiatives that promote the abilities of older people and their integration in the community. An example of this is intergenerational residential groups (10) and companionship schemes (14), in which older community members participate in mentorship and neighbourly roles. Such programmes have been shown to lead to positive quality of life outcomes in older individuals with dementia (15). In countries and cultures where intergenerational living is common, such as India, older people in intergenerational environments have been shown to have lower risks of short-term and chronic illness (16, 17).

We argue that fostering the intrinsic capacity and functional ability of older people will take longitudinal effort. To this end, intergenerational professional mentorship within health professional organisations, academia, and advocacy stakeholders is necessary to build the leadership capacities of a younger generation advocating for patient-centred care and healthy ageing. This mentorship should extend to the global health arena to ensure both the older and the younger generations progress in the global movement for a non-ageist world. We encourage the development of advocacy working groups for late-life health improvement, where older mentors liaise with youth participants to connect, cultivate leadership, and generate innovative intergenerationally-led solutions for ageing and health. These approaches are feasible for institutional actors to implement, and could take place within existing youth networks (e.g., the WHO's Global Health Workforce Network Youth Hub).

In the longer term, we encourage governments to devote resources towards training young researchers interested in inclusive age-related policy, mechanisms of ageing, ageing interventions, and/or functional improvements in later life. Over the past few decades, biogerontologists have made fundamental discoveries about the mechanisms of ageing (e.g., the relationship between metabolism, inflammation, and ageing). Devoting resources to early-career biogerontologists and implementation scientists will contribute to interest in translational projects as well as enhance the implementation of the fundamental discoveries in real-world settings over the coming decades. Additional research should consider the built environment and opportunities to create spaces appealing and accessible to all ages. Governments should enact policies that make environments physically more ageing-friendly. In all research, it is important to ensure that the perspectives of older adults are integrated.

## Delivering Age-Responsive Care

Third, *the Decade* calls for sufficient “delivery of person-centred integrated care and primary health services responsive to older people” (1). Our focus here is to promote the interest in and quality of care for older people. An inability to do so will contribute to the looming geriatrician shortage/geriatric care gap, and lead to care ignorant of evidence that links diseases of ageing to underlying aetiologies of ageing (18). Health professions schools should make clinical education on ageing and geriatrics mandatory, as called for by the International Federation of Medical Students' Associations, which represents the voice of 1.3 million medical students worldwide (19). This training should reflect developing scientific and social realities. Current clinical training on diseases of ageing is siloed into specialties with a single-disease focus, with trainees focusing on the treatment of diseases like cardiovascular disease, chronic obstructive pulmonary disease, and cancer, often not within the purview of geriatrics as currently configured. This weakens research and treatment capacity (20). Furthermore, clinical training about diseases of ageing needs to include training on the substantial links between patients' social realities and clinical outcomes.

Policymakers should meaningfully involve younger generations in discussions around how to strengthen primary health care and most effectively allocate resources to harness maximal economic potential. In addition to rethinking and reshaping health financing models to cover geriatric care at the primary care level, youth perspectives are crucial for the future planning of a health workforce which threatens to disappear if actions are not taken. For instance, medical students in Ireland led advocacy initiatives that called for increased funding for the ageing population and opposed the privatisation of nursing home care (21). Furthermore, these students orchestrated a day-long event in which local older people were able to share their concerns regarding healthcare and ageing with attending physicians and medical students. Such initiatives not only allow students to actively reflect on the type of systems in which they wish to work and age, but also provide a voice for older individuals who do not have direct access to communicating with the medical network. By uplifting their voices such that both older and younger people are advocating for change, there is increased amplification of the needs of older people.

## Providing Long-Term Care

Fourth, *the Decade* asks governments to “provide access to long-term care for older people who need it” (1). This is a necessary action area, but one that will likely place the responsibility of care on younger generations without adequate support for those carers. We call on governments to invest in young carers and caregivers through financial support and education as well as encourage solutions that engage young carers in service design (22). The intergenerational living programmes proposed earlier may be particularly effective in this respect, demonstrating how potential solutions to individual action areas are likely to positively impact multiple key sectors. Moreover, the rising demand for long-term care for older people amidst growing workforce shortages has negatively impacted aged care quality and safety efforts. Governments could involve youth in the co-creation of strategies to enhance the retention of the aged care workforce and mitigate shortages.

## Discussion

The journey into older age is a shared, inevitable human experience. Our response to *the Decade* proposal centres on how innovative intergenerational programmes, comprehensive and mandatory broad education about ageing, and the participatory role of youth in the global conversation on healthy ageing can concurrently address the four action areas of *the Decade*.

The desire to achieve these ideals and adequately address ageism amidst the grand challenge of our ageing society introduces a myriad of questions. How do we transfer best practice examples to increase their scope? How do we address subconscious ageism and ensure our efforts are truly centring on the wants and needs of older people? Indeed, how do we go beyond a needs-based approach and acknowledge that a cultural paradigm shift is needed that allows older people to not only have basic needs met but to also have wants, passions, and a valued place in society? How do we design a new social contract that benefits everyone, regardless of age?

## Author Contributions

BLHW led the writing of the manuscript. OE-O and DS provided guidance on youth engagement in global public health issues. DD, NA-C, and DG provided geriatric and gerontology-specific technical guidance. All authors contributed to the writing of the manuscript.

## Funding

DD was funded through a Wellcome Trust Intermediate Clinical Fellowship (WT107467) and DG through a Wellcome Trust Investigator Award (WT215574).

## Conflict of Interest

The authors declare that the research was conducted in the absence of any commercial or financial relationships that could be construed as a potential conflict of interest.

## Publisher's Note

All claims expressed in this article are solely those of the authors and do not necessarily represent those of their affiliated organizations, or those of the publisher, the editors and the reviewers. Any product that may be evaluated in this article, or claim that may be made by its manufacturer, is not guaranteed or endorsed by the publisher.
